# Bactericidal Pectin/Chitosan/Glycerol Films for Food Pack Coatings: A Critical Viewpoint

**DOI:** 10.3390/ijms21228663

**Published:** 2020-11-17

**Authors:** Bruno R. Machado, Suelen P. Facchi, Ariel C. de Oliveira, Cátia S. Nunes, Paulo R. Souza, Bruno H. Vilsinski, Ketul C. Popat, Mathew J. Kipper, Edvani C. Muniz, Alessandro F. Martins

**Affiliations:** 1 Laboratory of Materials, Macromolecules, and Composites (LaMMAC), Federal University of Technology—Paraná (UTFPR), Apucarana PR 86812-460, Brazil; brunomachado@alunos.utfpr.edu.br (B.R.M.); spfacchi@gmail.com (S.P.F.); ariel.colaco.aco@gmail.com (A.C.d.O.); quimicatia@hotmail.com (C.S.N.); 2 Group of Polymers and Composite Materials (GMPC), Department of Chemistry, State University of Maringá (UEM), Maringá PR 87020-900, Brazil; srpsouza1@gmail.com (P.R.S.); vilsinski@yahoo.com.br (B.H.V.); 3 School of Advanced Materials Discovery, Colorado State University (CSU), Fort Collins, CO 80523, USA; Ketul.Popat@colostate.edu (K.C.P.); matthew.kipper@colostate.edu (M.J.K.); 4 Department of Mechanical Engineering, Colorado State University (CSU), Fort Collins, CO 80523, USA; 5 Department of Chemical and Biological Engineering, Colorado State University (CSU), Fort Collins, CO 80523, USA; 6 Department of Chemistry, Federal University of Piauí, Teresina PI 64049-550, Brazil

**Keywords:** polysaccharides, antimicrobial, food packaging, coatings

## Abstract

Pectin and chitosan films containing glycerol (Gly) at 5, 10, 15, 20, 30, and 40 wt % were prepared in an aqueous HCl solution (0.10 M) by the solvent evaporation method. The unwashed film (UF) containing 40 wt % Gly (UF40) had elongation at break (*ε*, %) of 19%. Washed films (WFs) had high tensile strength (*σ* > 46 MPa) and low elongation at break (*ε*, <5.0%), enabling their use in food packaging applications. The polymers’ self-assembling occurred during the washing, increasing the stiffness. The XPS analysis suggests that some HCl is lost during the drying process, resulting in a low acid content on the UF surfaces. The UF40 (at 5.0 mg/mL) exhibits cytocompatibility toward mammalian cells and antimicrobial and anti-adhesive properties against *Escherichia coli*. The remaining HCl in the UF40 can be a disadvantage for food packaging applications; the UF40 (∅ = 8.5 mm; 55 μm thickness) releases H_3_O^+^/HCl, reducing the pH to approximately 3.0 when kept in 200 mL distilled water for approximately 30 min. Therefore, we propose the use of UF40 to coat commercial food packaging. The UF40 has low permeability to water vapor and oxygen and works as a barrier against ultraviolet light. The UF40 is also colorless and completely transparent. The UF40 maintained tomatoes’ structural integrity for 18 days at room temperature with no oxidation or microorganism contamination. This paper presents a critical viewpoint concerning chitosan-based films with antimicrobial activities.

## 1. Introduction

Bio-based materials can be used as food packaging materials because they can be cytocompatible, and biodegradable, and can replace the use of non-biodegradable, petroleum-based materials in the manufacturing of conventional plastics [1]. However, biodegradable-based films and coatings comprise only between 5% and 10% of the current plastics market. The principal issue limiting their widespread adoption is the high cost of production [2,3].

Polysaccharides have been used to develop food packages, including starch [4], cellulose [5], chitosan (CHT) [6,7], and alginate [8]. Chitosan-based films have advantages over other polysaccharide-based films because they can exhibit antimicrobial activity [7,9]. This feature can increase food’s shelf-life by preventing microbial growth.

CHT is a linear polysaccharide composed of random sequences of β-(1–4)-linked d-glucosamine and *N*-acetyl-d-glucosamine repeat units [10]. CHT is mainly obtained from partial alkaline deacetylation of chitin. Chitin is the second most abundant polysaccharide in the world, and it principally occurs in the exoskeletons of insects and crustaceans [11]. Therefore, CHT is obtained from an abundant, renewable, biodegradable, and inexpensive polysaccharide. This fact makes CHT a great candidate to comprise antimicrobial films [7,12] and coatings [13,14]. 

On the other hand, CHT has some disadvantages that include weak mechanical properties (requiring additional crosslinking [15]), high water vapor permeability [16], and low solubility in water and volatile organic solvents [17]. These features can discourage the use of CHT for manufacturing food packaging [18,19]. 

Effective moisture control is also essential for food preservation [20,21]. CHT is only soluble in aqueous solutions of dilute acids. Without effective washing and neutralization steps, residual acid can remain in the CHT-based films. The remaining acid can induce cytotoxicity (depending on the concentration) and certainly promotes antimicrobial activity. Indeed, some studies assign the antimicrobial activities of CHT-based films and coatings to the residual acid released from the materials [14]. Other studies attribute the antimicrobial properties to CHT only [12]. However, when CHT-based films and coatings are created from dilute acid solutions without washing and neutralization processes, the remaining acid should have antimicrobial effects. 

To overcome some of these disadvantages, we can blend CHT and pectin (PT) to make films, using the solvent evaporation approach [22,23,24]. There are some reports describing PT/CHT films [25,26,27]. In most cases, films have been created with an excess of CHT in the final material composition by blending the polymers in solutions with a pH from 3.0 to 6.0. The films obtained have weak mechanical properties for food packaging applications and usually quickly disintegrated in water [25,27,28].

When aqueous CHT solutions are combined with aqueous pectin (PT) solutions, polyelectrolyte complexes are formed by intermolecular interactions (coulombic and H-bonds) between the protonated amino groups (pK_a_ ≈ 6.0−6.5) on CHT and ionized carboxylic sites (pK_a_ ≈ 3.6−4.1) on PT [24,29,30]. Coulombic interactions between CHT and PT often generate materials with brittle structures and irregular surfaces when prepared at a pH from 3.0 to 6.0 [24]. In some cases, PT/CHT coacervates are yielded after blending the aqueous polyelectrolyte solutions [28,31]. The ionic strength, pH, temperature, and blend composition can all affect the properties of PT/CHT films and coacervates. 

PT/CHT blends in an aqueous dilute HCl (0.1 M, pH ≈ 1.0) solution, containing a highly *O*-methoxylated (56%) PT, produced durable and stiffer films (tensile strength between 18 and 40 MPa but with low elongation at break (approximately 2.0%), after the solvent evaporation and washing process to remove the residual HCl. The low pH (≈1.0) protonates the PT, preventing the uncontrolled association with the protonated CHT and precipitation of polyelectrolyte pairs (coacervates formation) in aqueous media. Due to the low elongation at break, these films cannot be used in food packaging applications. The PT/CHT film (at the ratio 66/34 wt/wt) was used as a scaffold for tissue engineering, which supported mammalian cells after 7 days of incubation [23,24]. 

There are no previous studies describing the use of food packaging composed of PT/CHT polyelectrolyte films plasticized with glycerol (Gly) in real applications. By tuning the Gly content in the PT/CHT blend, we create flexible films. Tripathi and coworkers created a PT/CHT/poly(vinyl alcohol) film for food packaging applications. The authors did not report mechanical property data; however, antimicrobial tests indicated action against *Escherichia coli*, *Staphylococcus aureus*, and *Bacillus subtilis* and antifungal properties against *Candida albicans* [12]. The authors pointed out that the film is suitable for food packing applications. However, this study did not demonstrate a practical application. Furthermore, this approach has disadvantages, including the use of synthetic poly(vinyl alcohol) as plasticizer (an expensive material) and an aqueous acetic acid (0.10 M) solution to solubilize the CHT with no washing step to remove the residual and volatile acetic acid. 

Here, we present a critical analysis of bactericidal PT/CHT polyelectrolyte films engineered for food packaging for the first time. We show that the antimicrobial properties depend on the residual acid content in the films. PT/CHT polymer blends were prepared in aqueous HCl (0.10 M). Gly (between 5.0 and 40 wt %) was added to blends before solvent evaporation. After solvent evaporation, the films were washed and dried for further study. We also selected unwashed films for further study. The washing process promotes the self-assembling of polymer chains, raising the tensile strength and reducing the elongation at break significantly. The washing step also neutralizes the residual H_3_O^+^/HCl in the films, attenuating their antimicrobial activities. The chemical, mechanical, optical, and surface properties of films (washed and unwashed) were characterized by XPS, SEM, mechanical and water contact angle measurements, thermogravimetric analysis, and absorption and transmission measurements. Due to the high stiffness, the washed films could not be applied as materials for food packaging. The XPS analysis confirms that a low HCl content remains in the unwashed film surface after solvent evaporation. The HCl is volatile and is evaporated during the drying step. We carried out cytotoxicity, antimicrobial activity, water vapor permeability, and oxygen permeability assays. The unwashed film containing 40 wt % Gly content (with elongation at break of 19%) was then used in a real application. The film prevented oxidation and microorganism adhesion on tomatoes for 18 days, increasing the food’s shelf-life compared with the commercial pack of polyvinyl chloride. However, when the unwashed circular film (∅ = 8.5 mm; 55 μm thickness) was in contact with water, it released H_3_O^+^/HCl, reducing the pH to approximately 3.0. We propose the PT/CHT film to coat conventional food packaging materials in low-humidity environments. 

## 2. Results and Discussion

### 2.1. Mechanical Properties

The mean thickness of the unwashed films (UFs) depended on the plasticizer content. It varied from 38 (±10) μm to 55 (±14) μm. The thickness increased as the Gly content increased in the polymer blend. On the other hand, the thickness of the washed films (WFs) was approximately 28 μm. The washing process should partly remove PT and Gly from the film structures due to their aqueous solubility. CHT should also be partly removed from the films in the washing step because they are prepared from polymer blends in an aqueous HCl solution (0.10 M). When the unwashed films were added to water (200 mL), the pH measured after 30 min was approximately 3.0. CHT is a cationic polysaccharide with solubility in dilute acid solutions due to its amino groups’ partial protonation. Protonated amino sites interact better with water molecules than non-protonated amines, supporting the solubility [24].

In the current study, we show that PT/CHT films (without plasticizer) with an excess of PT (66 wt %) in the polymer blend have high stability against dissolution/disintegration during the washing step. This finding was attributed to the high *O*-methoxylation degree on PT (56%) and the self-assembling of polymer networks throughout the washing step. *O*-Methoxylated moieties do not interact with water molecules like the carboxylic acid sites. Indeed, pectins of low *O*-methoxylation degrees have higher aqueous solubility than highly *O*-methoxylated pectins. In water, the carboxylic sites (pK_a_ ≈ 3.6–4.1) on PT ionize, interacting better with water molecules [23,24]. 

**Figure 1:**
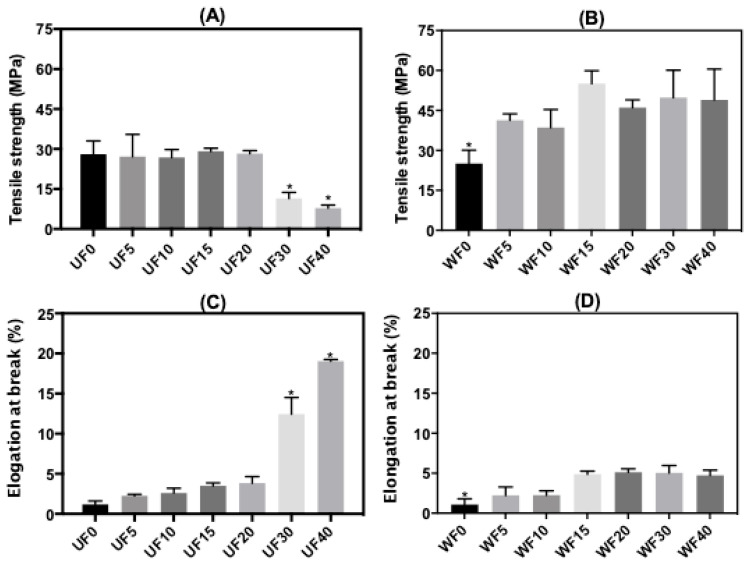
Tensile strength (*σ*, MPa, (**A**,**B**)) and elongation at break (*ε*, %, (**C**,**D**)) of the unwashed (UF) and washed (WF) films (dried samples) created from PT/CHT blends at different contents of glycerol (Gly) before solvent evaporation. (* indicates a statistically significant difference; *p* < 0.05).

Figure 1 presents the tensile strength (*σ*, MPa) and elongation at break (*ε*, %) of the PT/CHT/Gly films. Stress–strain curves of one replicate for each film are shown in Figure S1. The Gly content in the blends significantly changed the *σ* values. The mean *σ* values increased from 27 MPa (UF5) to 41 MPa (WF5), 26 MPa (UF10) to 38 MPa (WF10), 29 MPa (UF15) to 55 MPa (WF15), 28 MPa (UF20) to 43 MPa (WF20), 11 MPa (UF30) to 50 MPa (WF30), and 8.0 MPa (UF40) to 49 MPa (WF40) (Figure 1A,B). The increase in the Gly content from 5.0 to 20 wt % did not significantly change the value of *σ* for the UFs. This effect may be related to the low Gly concentration in the blend compared with the total polysaccharide mass [15]. Gly at 30 and 40 wt % significantly reduced the *σ* (*p* ≤ 0.05). In these cases, the Gly acted as a plasticizing agent in the films, reducing the *σ* (Figure 1A).

No significant increase occurred in the *σ* values as the Gly was added to the polymer blend after the washing step (*p* < 0.05). The washing should promote partial Gly removal from the films, resulting in higher *σ* values for WFs compared with the UFs (Figure 1B). However, it seems that the remaining Gly reinforces the WF structures. Low Gly contents can stabilize the films by the H-bonds established between Gly and polysaccharide networks. The Gly provides a plasticizing effect at 30 and 40 wt % only.

The results of elongation at break (*ε*, %) are shown in Figure 1C,D. The Gly (5.0, 10, 15, and 20 wt %) slightly increased the *ε* for the UFs compared with the WF0 (Figure 1C). Gly at 30 and 40 wt % significantly increased the *ε* values (*ε* = 12% for UF30 and *ε* = 19% for UF40, *p* ≤ 0.05). For the UFs at high Gly contents (30 or 40 wt %), the PT–CHT interactions reduced, increasing the films’ elasticity due to the plasticizing effect [15,32]. This behavior was also observed in the WFs; however, no significant difference in the *ε* values occurred even at a low Gly concentration (5.0 wt %). This finding must be related to the partial Gly removal from the WFs. The *ε* values for PT/CHT/Gly films were significantly different (*p* ≤ 0.05) concerning the *ε* for the FD0 (Figure 1D). This fact suggests that a low Gly content still remains in the WFs, reinforcing them. The stress–strain curves corroborated the *σ* and *ε* findings (Figure S1).

The Gly content in the polymer blend significantly influenced the mechanical properties. The average *ε* values varied from 2.0 to 19%, while the *σ* was between 8.0 and 55 MPa. These findings agreed with other results. For instance, PT/CHT films incorporated with surfactant (Tween 80) created from the solvent evaporation approach without a plasticizer, but with citric acid as a crosslinking agent, presented *ε* values between 1.0 and 6.5% and *σ* between 22 and 44 MPa [26].

### 2.2. X-ray Photoelectron Spectroscopy

**Figure 2:**
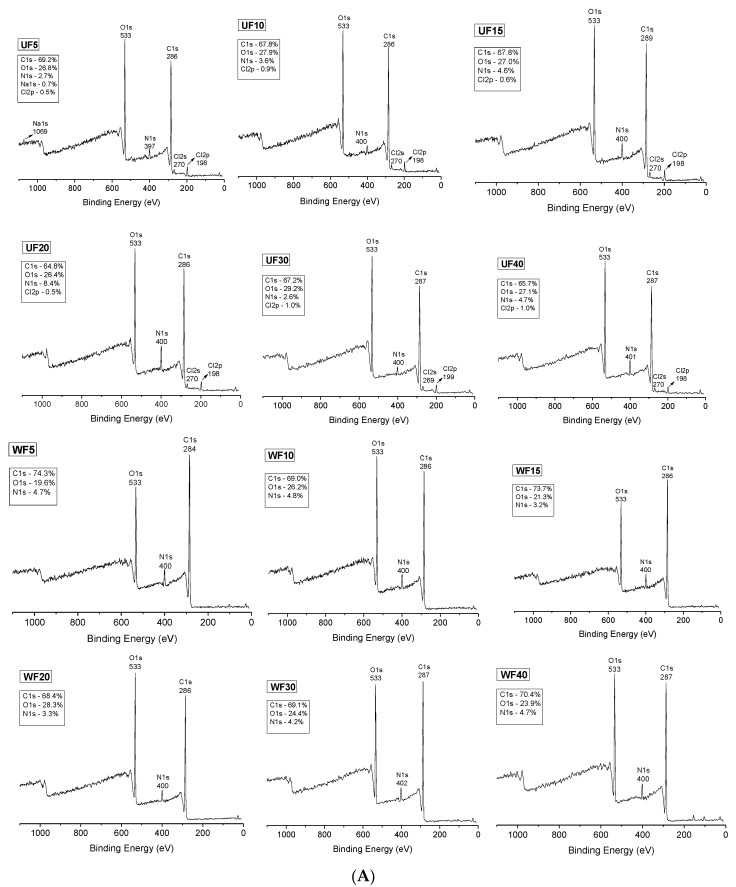

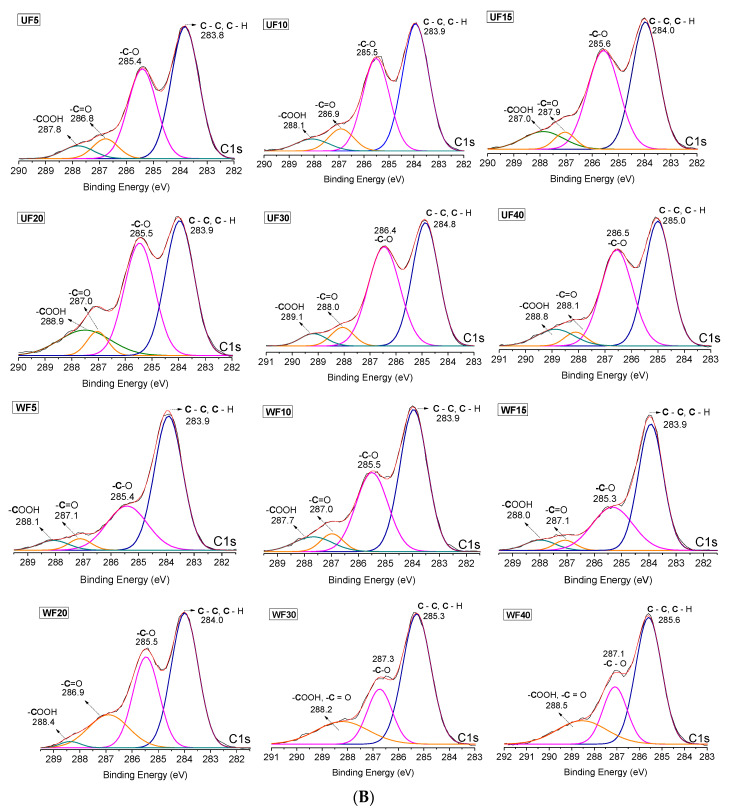
(**A**) Survey XPS spectra of the unwashed (UF) and washed (WF) films created from PT/CHT blends containing different Gly contents before solvent evaporation. (**B**) High-resolution XPS spectra of the unwashed (UF) and washed (WF) films created from PT/CHT blends containing different Gly contents before solvent evaporation: C_1s_ envelopes.

The chemical composition of the films’ surfaces was analyzed by XPS. XPS (survey) spectra are shown in Figure 2A. All XPS spectra show characteristic peaks assigned to oxygen (O_1s_, 533 eV) and carbon (C_1s_, 284–289 eV) [33]. These elements occur in the chemical structures of PT, CHT, and Gly. The spectra for CHT also contain the characteristic signal of nitrogen (N_1s_, 397–401 eV). The presence of chlorine (Cl_2p_, 198–199 eV) on the UFs was related to the aqueous HCl solution used to create the polymer blends. Even before washing, the Cl_2p_ content was low (between 0.5 and 1.0%, Figure 2A) on the UF surfaces. Thus, the HCl in the blends must be evaporated in the drying process, reducing the UF surfaces’ Cl_2p_ content. After washing, the residual HCl on the WF surfaces was completely removed because the XPS spectra do not indicate Cl_2p_ peaks (Figure 2A).

The N_1s_ content on the UF surfaces altered from 2.6% to 8.4% (Figure 2A). The concentration depends on the film area analyzed in the XPS. We emphasize that the films were created at 34 wt % CHT and 66 wt % PT. The survey XPS spectra also indicate an alteration in the N_1s_ content (between 3.2% and 4.8%) on the WF surfaces (Figure 2A). However, the variation in the N_1s_ content was smaller on the WF surfaces than on the UF surfaces. The washing process should reorganize the polymer chains for more stable configurations. After washing, the N_1s_ content is between 3.0% and 5.0%. The structural reorganization of the polymer chains will be confirmed by thermal analysis.

On both UF and WF surfaces, the C_1s_ and O_1s_ contents are similar, with C_1s_ between 64.8% and 69.2% and O_1s_ between 26.4% and 29.2%. PT, CHT, and Gly are mostly composed of carbon and oxygen. After washing, the C_1s_ and O_1s_ contents also changed, varying between 68.4% and 74.3% for the C_1s_ and between 19.6% and 28.3% for the O_1s_.

The washed PT/CHT film created at a ratio of 67/23 wt/wt had elemental compositions of C_1s_ = 71.5%, O_1s_ = 23.9%, and N_1s_ = 3.7% [23]. We then confirmed that the Gly was removed from the WF surfaces during the washing. However, the mechanical measurements indicate that a low Gly content should occur in the WFs, reinforcing them. Therefore, a small amount of Gly remained in the film structures even after washing. Comparatively, the C_1s_ content increased, while the O_1s_ level decreased after washing. This effect is due to Gly removal from the WF surfaces. The high-resolution XPS spectra of the C_1s_ envelopes confirm this finding (Figure 2B).

The high-resolution XPS spectra confirm the presence of characteristic chemical groups related to the polysaccharide structures and Gly. We report the peaks assigned to–C–C and –C–H (283–286 eV), –C–O (285–288 eV), –C=O (286–289 eV), and –COOH (287–289 eV) [23,34].

The high-resolution XPS spectra (C_1s_) of the UFs present –C–O peaks with high intensities due to the presence of Gly. This effect is evident with the 20 wt % Gly concentration (Figure 2B). After washing, the –C–O peaks present low intensities, indicating Gly removal from the WF surfaces. The C_1s_ envelopes obtained for WF30 and WF40 are different from the others. The peaks ascribed to the chemical –COOH and –C=O groups overlapped (Figure 2B). This effect is related to the structural reorganization of the polymer chains during the washing process.

The films were also characterized by SEM (Figure S2). The films show compact structures with no large pores. Smooth surfaces seem to occur at high Gly contents.

### 2.3. Thermal Analysis

**Figure 3:**
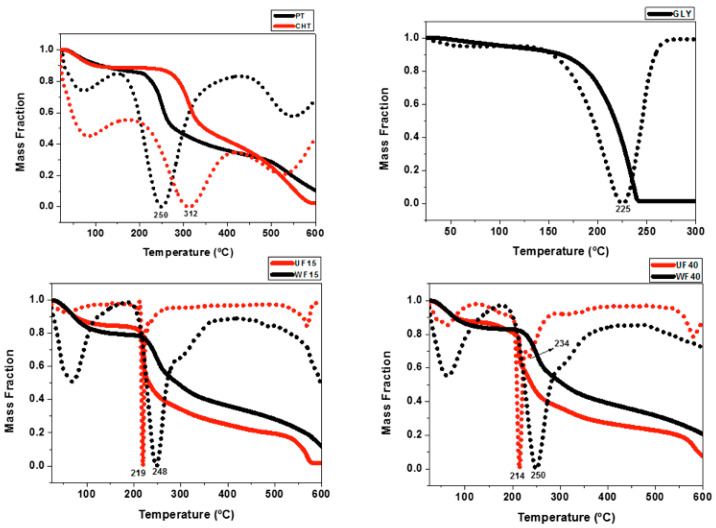
TGA (solid lines) and DTG (dotted lines) curves of the polymer precursors (PT and CHT), glycerol (Gly), and PT/CHT films.

TGA and the first derivative of the thermogravimetric curves (DTG) of the precursors and films are presented in Figure 3. PT and CHT show three main events in the TGA/DTG profiles. The first, between 40 and 150 °C, is related to the loss of water and volatile compounds. The second, between 150 and 450 °C, is assigned to the degradation of polymer chains owing to pyrolytic decomposition. The third event, between 450 and 600 °C, is associated with the degradation of by-products [34]. The Gly TGA curve indicates thermal degradation between 150 and 250 °C [35].

TGA/DTG curves of the UFs (UF15 and UF40) and WFs (WF15 and WF40) suggest similar water and volatile compound contents (between 16% and 20%). However, the second thermal event related to degradation occurs at higher temperatures for the WFs. The UF40 TGA curve presents an additional event at 234 °C. Probably, this event occurs due to the presence of Gly (Figure 3). The WFs show high thermal stability due to structural reorganization of the materials in the films. This finding corroborates with the mechanical property measurements, indicating that the films’ components interact more between each other in the WFs than in the UFs.

A previous study showed that PT/CHT films (UF0 and WF0, without plasticizer) have degradation temperatures of around 235 and 253 °C, respectively [24]. Comparing the UF0 with the UF15 and UF40, we show that Gly decreases the thermal stability, achieving 16 °C for UF15 and 21 °C for UF40. The degradation temperature reduced as the Gly concentration increased. Xu and coworkers showed that CHT/hemicellulose films degraded at 169 °C, while the film with 40% Gly degraded at 139 °C [32]. On the other hand, the WF0, WF15, and WF40 presented similar TGA profiles due to Gly removal during the washing process. The inflection points in the DTG curves occur at 253 °C (WF0 [24]), 248 °C (WF15), and 250 °C (WF40). These findings suggest structural reorganization of the films and Gly removal due to the washing step.

### 2.4. Water Contact Angles

Water contact angle (WCA) measurements were carried out on the films prepared with 15, 30, and 40% Gly content in the polymer blends (Table 1). Figure S3 presents digital images of the film surfaces with a water droplet. The main objective of this analysis was to evaluate the wettability of the films after plasticizer incorporation and the washing step.

The surface wettability increased as Gly was added to the polymer blend. At time *t* = 0, all films were hydrophobic (WCA higher than 90°). However, after 10 min, the WCA for UF15, UF30, and UF40 reduced to 72°, 63°, and 50°, respectively. The water droplet interacts with Gly molecules through H-bonds, spreading on the film surface [32]. The WFs present higher WCAs than the UFs due to Gly removal during the washing process. The WCAs are 89° (WF15), 78° (WF30), and 77° (WF40) after water droplet contact at 10 min (Table 1).

The washing process significantly alters the films’ properties. The water droplet absorption is slower on the WFs than on the UFs. The Gly removal produces stiffer materials, preventing fast water droplet absorption. This result agrees with the tensile strength measurements, which indicated high values (between 48 and 55 MPa) for the WFs.

The washing promotes the self-assembling of polymer chains, altering the surface wettability. Stiffer films should take a longer time to absorb a water droplet than elastic films. Thus, the WFs show higher WCAs than the UFs. These results also agree with the XPS data, which indicates Gly removal from the film surfaces in the washing step. The WCAs on WFs (WF15, WF30, and WF40) are similar to the WCA determined on WF0 (97° after *t* = 0 min and 79° after *t* = 10 min) [23].

Our results agree with other findings. Citral essential oil/PT/alginate/Gly films had WCAs between 35° and 49° [36]. The presence of essential oil increased the WCAs. Other PT/CHT films (weight ratios at 75/25, 50/50, and 25/75) had WCAs between 74° and 88°. The WCAs increased at higher CHT contents.

### 2.5. Light Absorption and Transmittance

Based on the mechanical properties, we selected the UF40 (with the highest *ε*, (19%)) and WF15 (with the highest *σ*, 55 MPa) samples for further study. For a better analysis, we compared the light barrier properties of the UF40 and UF15 samples with the WF40 and WF15 samples, respectively. Food packaging should block ultraviolet light to prevent oxidation of the contents. Lipid oxidation significantly influences the organoleptic properties and reduces the food’s shelf-life. Film barriers can delay the oxidation of lipids, protecting the food against degradation [20].

**Figure 4:**
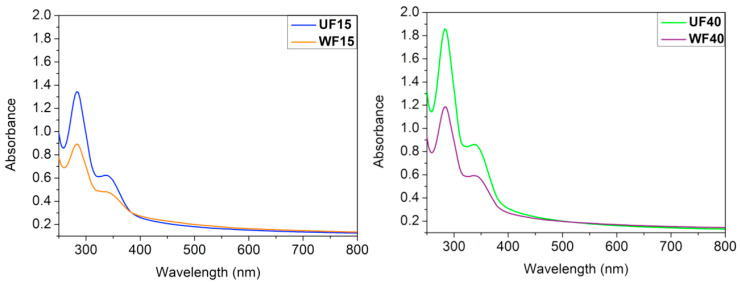
UV-Vis spectra of the unwashed (UF15 and UF40) and washed (WF15 and WF40) films created from a PT/CHT blend at 15 and 40 wt % Gly content.

UV-Vis absorption spectra of the films are presented in Figure 4. The UFs have greater absorbance than the WFs. The Gly in the UFs can absorb UV light. The films’ thickness also influences the light barrier capacity. The WFs’ thickness (WF15 = 0.028 mm and WF40 = 0.029 mm) is less than the respective UFs’ thickness (UF15 = 0.038 mm and UF40 = 0.055 mm, Table 2). Therefore, the UF40 shows the highest absorbance in the ultraviolet range (Figure 4). When the Gly content increases from 15% to 40%, the protective effect is still more enhanced due to the greater film thickness and Gly’s capacity to absorb UV light [37].

Film transparency is an essential factor for manufacturing food packaging. This effect has a direct impact on consumer acceptability [37]. The transparency results in Table 2 can confirm that the WF films are more transparent at λ = 600 nm.

The UF40 has the lowest transparency, indicating that this film adsorbs more light than the other samples. The low transparency is intimately related to the high thickness [38]. The UF40′s transparency (34.80 %·mm^−1^) is similar to the transparency of polypropylene (38.20 %·mm^−1^) and higher than low-density polyethylene’s transparency (between 15 and 20 %·mm^−1^) [39].

Similar to our results, Cazon and coworkers showed that poly (vinyl alcohol)/cellulose/Gly films have low transparency and absorb UV light as the Gly content is increased in the film. The highest transparency (89.38 %·mm^−1^) was achieved at 3.0% wt/wt cellulose, 5.0% wt/wt Gly, and 5.0% wt/wt poly(vinyl alcohol) [40]. Tan and coworkers developed CHT films with grapefruit seed extract for bread packaging [41]. The light barrier protection rose from 58.84 to 93.14%·mm^−1^) as the fruit extract concentration increased from 0 to 1.5% wt/wt. Commercial packaging, including syndiotactic polypropylene (89.1%·mm^−1^), polyester (83.5%·mm^−1^), and poly(vinyl vinylidene chloride) (90.0%·mm^−1^) have similar properties [41].

### 2.6. Permeability to Water Vapor and Oxygen

Water vapor and oxygen permeability are the main investigated properties of films used in real applications—water vapor and oxygen permeability influence food’s shelf-life [20]. Food packaging should reduce the water vapor permeability to prevent microbial growth on food. Food packaging should also prevent contact with oxygen because it oxidizes the food, causing degradation. The findings of water vapor and oxygen permeability on the samples UF40 and WF15 are shown in Table 3.

Gly at 40 wt % imparts low water permeability and high oxygen transmission to the UF40. The hydrophilic Gly interacts with water molecules, reducing the water vapor permeability. On the other hand, oxygen transmission is high through the UF40 due to the oxygen gas’s hydrophobicity. The hydrophobic WF15 interacts better with non-polar oxygen molecules, reducing their transmission (Table 3).

These results agree with the WCA measurements. The high UF40 hydrophilicity (WCA = 50° after 10 min) compared with the WF15 (WCA = 89° after 10 min) reduces the water vapor transmission. A high Gly content in the UF40 reduces oxygen transmission. Therefore, the plasticizer concentration plays an essential role in the barrier properties [42].

### 2.7. Cytotoxicity

The cytotoxicity was determined by LDH assay. LDH is an enzyme found in the cytoplasm. It is released from the cells as the membrane integrity is disrupted. LDH acts as a marker of physical cell integrity [43].

The cytotoxicity (%) findings were normalized using the results obtained on polystyrene (PS, negative control) and in an aqueous Triton X solution at 1.0 vol % (positive control), following the manufacturer’s protocol. The experiment with the PS control verified the spontaneous LDH release because it promotes cytocompatible food packaging [44,45]. The cells were treated with Triton X, which permeabilizes the membrane providing total LDH release [23].

The cytotoxicity was investigated against human ADSCs for 24 h. The UF40 and WF15 promoted cytotoxicity of 5.5% and 3.5%, respectively (*p* > 0.95). Both films exhibited very low percentages of cytotoxicity. We highlight that even the UF40 was cytocompatible for the ADSCs at approximately 5.0 mg/mL. Such an effect is due to the low HCl concentration in the UF40 or the low concentration (5.0 mg/mL) used in the cytotoxic test.

The cytotoxicity test was carried out in a cell culture medium at a pH close to 7.4. The medium should also neutralize the remaining HCl in the UF40, reducing the cytotoxicity to mammalian cells. The LDH assay already showed that pure PT/CHT films without Gly are cytocompatible with ADSC cells [23].

Sterilization with ethylene oxide can promote methylation of the –NH_2_ sites on CHT chains. However, this effect should be attenuated when the process is carried out at 40 °C for 120 min compared with longer processes of around 8 h at 40 °C [46]. Methylated amine moieties can induce cytotoxicity; however, we suggest that the films are cytocompatible.

### 2.8. Antimicrobial Properties

Antimicrobial tests with the UF40 and WF15 were evaluated against *E. coli*, a pathogenic bacterium associated with food contamination and alimentary intoxication [47,48]. Film disks (8.0 mm at ≈5.0 mg/mL) were added to Petri dishes containing *E. coli* for 24 h (Figure S4). There was no formation of inhibition halos, even using UF40 disks. We conclude that there is no release of H_3_O^+^/HCl from the UF40 during the antimicrobial test carried out by the disk diffusion method. However, *E. coli* cells do not grow on the UF40 surface as they do on the WF15 surface (Figure S4). It seems that the UF40 surface provides an anti-adhesive effect, preventing adhesion of *E. coli* cells.

**Figure 5:**
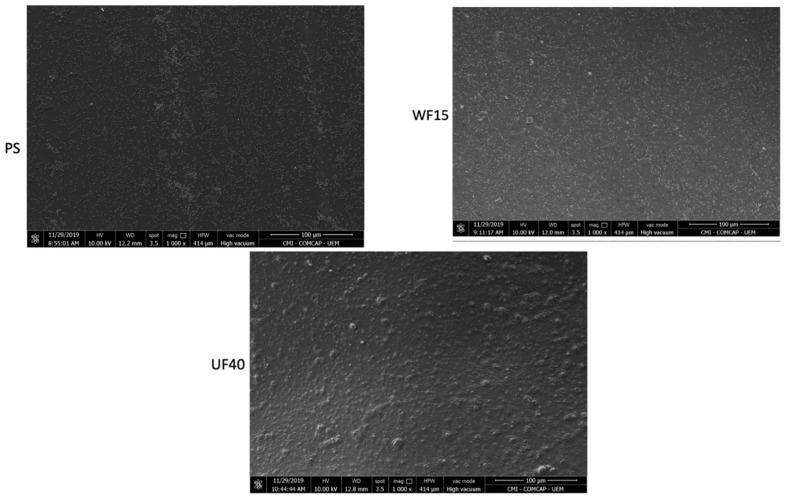

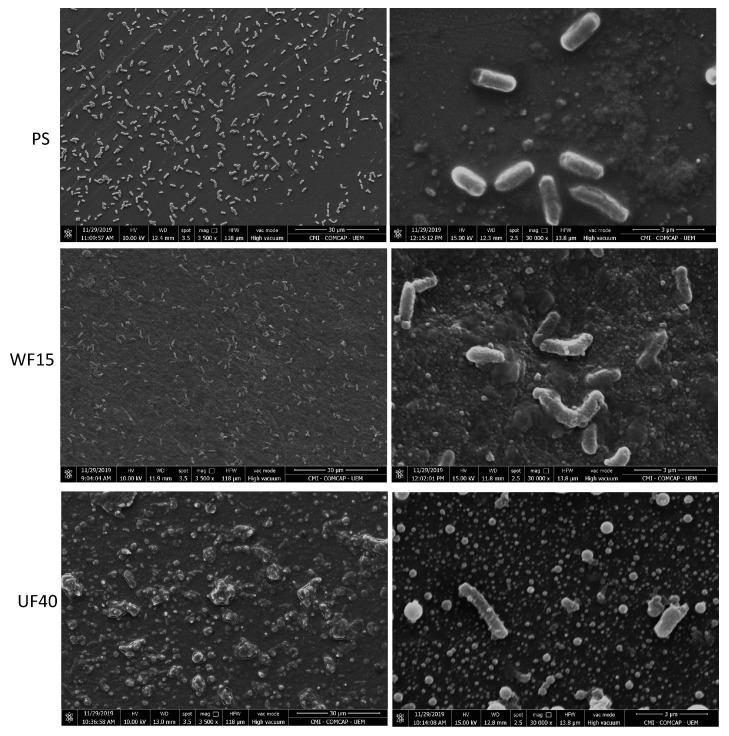
SEM images of the polystyrene (PS, control), WF15, and UF40 incubated with *E. coli* after 24 h of exposure. SEM images at 1000× (scale bars = 100 μm), 3500× (scale bars = 30 μm), and 30000× (scale bars = 3 μm).

Therefore, we performed anti-adhesive and proliferation tests by seeding *E. coli* cells with the films for 24 h. SEM images show no adhesion of microbial cells with regular morphology onto the UF40 surface (Figure 5). On the other hand, we found *E. coli* cells with normal integrity on the WF15 and PS surfaces. The UF40 has anti-adhesive activity against *E. coli*. These findings agreed with the outcomes reported in the disk diffusion approach. The PS was selected as a negative control because it does not have antimicrobial and anti-adhesive activities, and it is also used in food packaging applications [44,45]. As expected, bacterial cells with regular morphology completely covered the PS surface after 24 h of exposure (Figure 5).

The anti-adhesive capacity is supported by the UF40 results regarding its higher surface wettability than the WF15 and PS (WCA = 82° [49]). The cell walls of Gram-negative bacteria are coated with a thin layer of glycoproteins and covered by a thick layer, mainly composed of lipoproteins and lipids. In this way, films and coatings with hydrophobic surfaces should interact better with the microbial cells, favoring adhesion. Hydrophilic surfaces should prevent the attachment of *E. coli* cells [13,14,49,50].

The UF40 also has bactericidal activity. The bactericidal action is related to the cationic –NH_3_^+^ group in CHT. The protonated amino group electrostatically interacts with the phospholipid dipalmitoyl phosphatidylglycerol (DPPG), the main component of Gram-negative cell membranes. Such an interaction increases the cell membrane permeability, causing evasion of intracellular components, such as nucleic acids and glucose, and preventing the transport of nutrients to the microbial cells. These events promote cell death [13,51]. These results agree with other findings. Soni and coworkers showed that the antimicrobial action of CHT/2,2,6,6-tetramethylpiperidine-1-oxyl films against *E. coli* and *Salmonella* depended on the CHT concentration in the film. A pure CHT film promoted the highest antimicrobial activity at pH conditions lower than 6.3 due to the presence of –NH_3_^+^ groups on the CHT chains [52]. However, the pH conditions in which the film was created play a critical role in the antimicrobial properties. After contact with the cell culture medium, the UF40 film swells and releases H_3_O^+^ and HCl, killing the microbial cells. Thin films based on chitosan/heparin assembled at pH 2.9 have higher bactericidal and anti-adhesive properties against *E. coli* cells than the polymer assemblies created at pHs higher than 4.0 [14].

CHT/pectin and CHT/*iota*-carrageenan thin films assembled in an aqueous acetic acid/sodium acetate solution (0.20 M at pH 5.0) also had anti-adhesive and antimicrobial properties against *Staphylococcus aureus* (Gram-positive bacterium) and *Pseudomonas aeruginosa* (Gram-negative bacterium) [13]. Therefore, CHT-based films and coatings foster intense antimicrobial activities due to the synergistic effect among the pH condition (used to create the material), the release of residual acid from the material surface, the presence of cationic –NH_3_^+^ sites on the material surface, and surface wettability.

### 2.9. Food Package Application

We have shown that the UF40 (disks of 8.0 mm at ≈5.0 mg/mL) have no cytotoxicity and, depending on the preparation, can exhibit antimicrobial activities. WFs have no potential to be applied as food packaging materials due to their low flexibility and high stiffness. Here, we selected an aqueous HCl solution (0.10 M) to dissolve both CHT and PT and create miscible mixtures. We tried to use aqueous citric and acetic acid solutions (0.10 M) to dissolve the polysaccharides at pHs higher than 2.5. However, polysaccharide blends were not created, leading to polyelectrolyte precipitates. In this case, PT (pK_a_ ≈ 3.6–4.1) and CHT (pK_a_ ≈ 6.0–6.5) interacted mainly by electrostatic interactions, forming conventional coacervates. CHT is not soluble in aqueous solutions at a pH higher than 6.0. Therefore, we justify using an aqueous HCl solution to form PT/CHT blends in a low pH condition to avoid coacervate formation [28,31].

**Figure 6:**
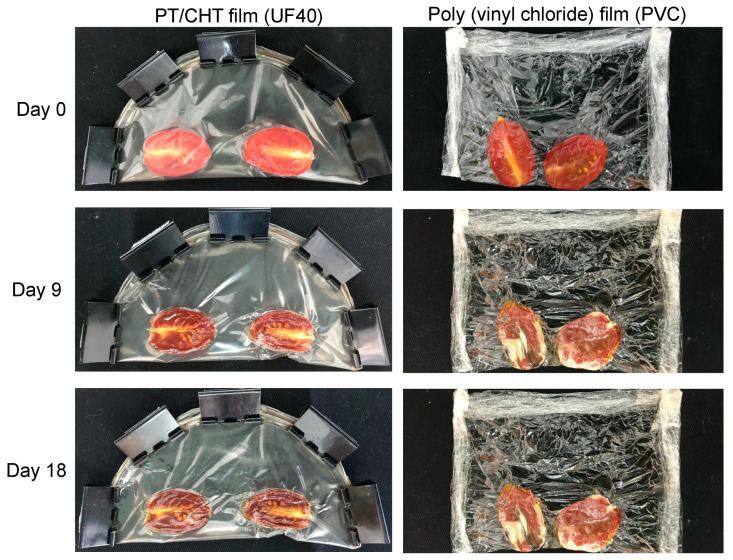
Tomatoes exposed to the UF40 and commercial poly (vinyl chloride) packs after 0, 9 and 18 days.

As a proof of concept, for a real application, we tested the UF40 pack (a film disk with 15 cm diameter) for tomatoes because of its high elasticity, cytocompatibility, and antimicrobial activities at ≈5.0 mg/mL. Figure 6 shows digital images of tomatoes covered by the UF40 and, for the sake of comparison, with polyvinyl chloride after storage for 0, 9, and 18 days at room temperature.

The tomatoes present their characteristic red color and smooth texture at Day 0 (Figure 6). After 9 days of storage, their physical appearance altered. The UF40 dehydrated the tomatoes, preserving them. The UF40 is a hydrogel film; therefore, it can absorb water from the food. The UF40 also prevented contamination by microorganisms, improving the preservation of the tomatoes, compared with the commercial polyvinyl chloride packaging. The commercial packaging did not prevent microorganisms that grew in the central fruit tissues. The polyvinyl chloride did not protect the food after 9 and 18 days of storage. The UF40 conserved the tomatoes for until 18 days at room temperature, as clearly seen in the photo.

## 3. Conclusions

This study characterizes pectin/chitosan (PT/CHT) films incorporated with different glycerol (Gly) contents (between 5.0 and 40 wt %). The films are proposed for food packaging applications. We used an aqueous HCl solution to create the polymer blends and produced films by the solvent evaporation approach. We characterized the chemical, mechanical, and physical properties of the films by mechanical analysis, XPS, SEM, water contact angle measurements, and TGA/DTG analysis. Unwashed (UF) and washed (WF) films were created. Washed films (WFs) have higher stiffness and cannot be used as food packs due to their low elasticity. The washing process removes the Gly from the films. The unwashed films (UFs) have low residual HCl content in their structures, which was confirmed by XPS. Gly at 40 wt % creates a flexible film (UF40) with elongation at break of 19%. We selected the UF40 for further study because it has antimicrobial and anti-adhesive properties against *E. coli* at approximately 5.0 mg/mL. *E. coli* is a pathogenic bacterium responsible for food contamination.

It is challenging to create food packs based on PT/CHT polyelectrolyte complexes. CHT-based materials often have weak mechanical properties. Aqueous mixtures of polyanionic and polycationic polymers usually support coacervates as well. We produced miscible PT and CHT mixtures, using an aqueous HCl solution (0.10 M at pH ≈ 1.0) as a solvent. The residual HCl in the film may promote undesirable traits for food packaging applications. Therefore, we propose the UF40 to act as a coating material for conventional packs. UF40 can significantly increase food’s shelf-life.

This work improves upon the use of bactericidal CHT-based films for food packaging applications. CHT packaging cannot be created without residual acid content in their structures. The residual acid is the principal agent responsible for antimicrobial activity. Here, we show that the UF40 (composed of PT, CHT, Gly, and a low level of residual HCl) has suitable mechanical properties and antimicrobial capacity against *E. coli*. An additional step of washing alters the films’ traits, increasing the stiffness due to polymer self-assembly. After solvent evaporation, the UF40 in water (200 mL) results in a pH of approximately 3.0. Depending on the application, this pH can provide cytotoxicity. After water contact, UF40 released H_3_O^+^/HCl, reducing the pH. We conclude that the acid used to create CHT-based solutions imparts antimicrobial properties to the films.

## 4. Materials and Methods

### 4.1. Materials

GENU^®^ pectin (PT) with a molar mass of 190 kDa and an *O*-methoxylation degree of 56% (extracted from orange peels) was graciously donated by CP Kelco (Limeira, Brazil). Chitosan (CHT) with a deacetylation degree of 85% and a molar mass of 87 kDa was acquired from Golden-Shell Biochemical (Zhejiang, China). Glycerol (99%, Gly) was obtained from Sigma-Aldrich (São Paulo, Brazil).

### 4.2. Preparing the Films

Films were created by following experimental procedures reported elsewhere [22,23,24] with modifications. Aqueous PT and CHT solutions (1.0% wt/vol) were separately prepared in an aqueous HCl solution (0.10 M) at 60 ± 1 °C for 10 min. Polymer blends were prepared at 60 ± 1 °C for 5.0 min, using the pre-formed solutions at 1.0% wt/vol. The PT/CHT weight ratio in the blend was equal to approximately 66/34. Different Gly contents were then added to the polymer blend solution (30 mL) at 60 ± 1 °C. The Gly content (wt %) was calculated based on the total polymer mass (PT + CHT = 0.30 g) in the blend. Table 4 shows the experimental conditions used for film preparation. After Gly addition, each formulation was stirred for 5.0 min at 60 ± 1 °C, and the resulting solution was added to Petri dishes (∅ = 8.5 mm; 10 mm thickness). The solvent (aqueous HCl solution at 0.10 M) was evaporated in the oven at 35 ± 1 °C for 24 h. The films prepared using different Gly contents were peeled off the Petri dishes and stored at room temperature for further analysis. Additional PT/CHT/Gly films peeled off the Petri dish were subsequently washed by soaking in deionized water (200 mL) at 25 ± 1 °C. An aqueous NaOH solution (0.010 M) was slowly dripped into the system over 6 h, to raise the pH to approximately 5.5. After the washing step, the films were oven-dried at 35 ± 1 °C for 24 h and stored at room temperature for further analysis. The washed films are labeled as WFx while the unwashed ones are labeled as UFx, where x is the Gly content (wt %) added to the PT/CHT blend before solvent evaporation (Table 4).

### 4.3. Characterization

The films’ thickness was determined in 50 × 25 mm samples (*n* = 5), using a digital micro-durometer (model ZAAS). The mechanical properties, including tensile strength (*σ*, MPa), elongation at break (*ε*, %), and stress–strain curves, were determined using a MicroSystems texture analyzer (model TATX2i, England), according to the ASTM D882-02 standard (*n* = 5) [53]. Dried samples (50 × 25 mm) were stored in a saturated Mg(NO_3_)_2_ solution at 25 ± 2 °C for 48 h and a relative humidity of 53% ± 2%. The crosshead speed was regulated to 0.83 mm/s (load cell of 50 kg) with an initial distance of 30 mm between the grids.

The surface chemistry of the films was assessed using a Phi Electronics 5800 spectrometer (Chanhassen, MN, USA). X-Ray photoelectron spectroscopy (XPS) spectra were obtained with a monochromatic AlKα X-ray source (*hν* = 1486.6 eV), with a hemispherical analyzer and a multichannel detector. High-resolution XPS spectra were obtained at 23.5 eV analyzer pass energy with 0.1 eV steps and an X-ray spot of 800 μm. The take-off angle of 45° was used for all spectra. Charge neutralization was achieved using a low-energy electron gun. Origin 8.5 software was used to perform the fitting in the spectra curves. Gaussian peaks were fitted according to the expected functional groups.

Water contact angles (WCAs) were determined on the film surfaces, using the sessile-drop approach with a Krüss DAS 10 goniometer (Hamburg, Germany). Water droplets (5 μL) were deposited on the films and WCAs were measured at 0 and 10 min. WCAs were monitored for 10 min using DAS 10 software (*n* = 3).

Thermogravimetric analysis (TGA) was carried out in a Shimadzu thermogravimetric analyzer (model TGA-50, Japan) at a 10 °C/min rate under a constant argon purge (50 mL/min). The morphology was investigated using scanning electron microscopy (SEM) (model JSM-6500F, JEOL, Japan) using an accelerating voltage of 5 kV. To prepare for SEM imaging, the samples were sputter-coated with a gold–palladium alloy at a thickness of 10 nm.

### 4.4. Light Absorbance and Transparency

The films’ transparency was determined by following a reported experimental procedure [40]. The analysis was performed with a spectrophotometer (Perkin Elmer, Lambda 750, USA) in the absorbance mode (*n* = 2). Absorbance was measured using film samples (1.0 × 1.0 cm) over the wavelength range from 250 to 800 nm. The transmittance (*T*_600_) at 600 nm was used to determine the films’ transparency using Equation (1).

(1) $$Transparency\;=\;\frac{log\%T_{600}}{X}$$

where %*T*_600_ is the transmittance percentage at 600 nm and *X* is the film thickness (mm).

### 4.5. Water Vapor and Oxygen Permeability

The water vapor permeability was measured in a Permatran apparatus (model W3/3, Mocon, France), following the ASTM F1249-90 standard, using a modulated infrared sensor [54]. Experiments were conducted at a relative humidity of 90%, with a calibration standard of 5.21 g·m^2^/day at 23 ± 1 °C. The oxygen permeability was measured in an Oxtran apparatus (Mocon, France), coupled with a colorimetric sensor, according to the ASTM D3985-02 standard [55]. These analyses were performed by Amcor Flexibles Latin America.

### 4.6. Cytotoxicity

Human adipose-derived stem cells (ADSC cells) were cultivated in Dulbecco’s Modified Eagle Medium (DMEM) with 10% fetal bovine serum and 1.0% penicillin/streptomycin at 37 ± 1 °C and 5% CO_2_. Cells of Passage 2 were used. Before seeding the cells, film disks (8.0 mm; weights of approximately 2.5 mg for UFs and 1.7 mg for WFs) were fixed with carbon tape on Teflon disks. The samples were then sterilized with ethylene oxide through the alkylation process at 40 ± 1 °C for 120 min [46]. The films’ cytotoxicity was evaluated using the commercial lactate dehydrogenase (LDH) cytotoxicity assay kit, according to the manufacturer’s instructions.

ADSC cells (15,000 cells/well) were cultivated in 48-well plates in the presence of films, polystyrene disks (8.0 mm, negative control), and Triton X solution (1.0 vol%, positive control). After 24 h of incubation, the activity of LDH released in the cell culture medium was determined and the cytotoxicity (%) was calculated using Equation (2):

(2) $$Cytotoxicity\;\left(\%\right)=\;\frac{\left(LDHa-LDHe\right)}{\left(LDHt-LDHe\right)}\times 100\;$$

where *LDHa* is the LDH activity promoted by the films, *LDHe* is the spontaneous LDH activity in the presence of polystyrene, and *LDHt* is the maximum LDH activity promoted by the Triton X at 1.0 vol% (*n* = 5).

### 4.7. Antimicrobial Assay

The samples were sterilized by ethylene oxide at 40 ± 1 °C for 120 min [46]. The antimicrobial assay was carried out according to the Clinical and Laboratory Standards Institute [56,57]. The antimicrobial assay was performed against the Gram-negative bacterium *Escherichia coli* (*E. coli*, ATCC 25922) in Brain Heart Infusion (BHI) after incubation for 24 h at 37 ± 1 °C. The assay was conducted in sterilized tubes containing saline solutions (0.85% wt/vol) at 1.0 × 10^7^ CFU/mL). *E. coli* suspensions (500 μL at 1.0 × 10^7^ CFU/mL) in Müeller–Hinton agar (37 ± 1 °C for 24 h) were added to the film disks (8.0 mm; weights of approximately 2.5 mg for UFs and 1.7 mg for WFs) fixed with carbon tapes in 48-well plates. After 24 h, the samples were removed from the plates and fixed with a glutaraldehyde solution (3.0% wt/vol) containing sodium cacodylate (0.10 M) and sucrose (0.10 M) for 45 min at room temperature. After fixing, samples were washed with deionized water, frozen, and lyophilized. SEM images of the film surfaces were obtained at 10 and 15 kV for the cellular morphological analysis of *E. coli* adhering to the films. Three samples were considered for each film (*n* = 3). Polystyrene disks were used as a control. We also evaluated the antimicrobial activities by performing the disk diffusion method as reported elsewhere [58].

### 4.8. Food Package Application

For this analysis, a polymer solution (90 mL) composed of PT, CHT (PT + CHT = 0.9 mg, PT/CHT weight ratio at 66/34), and Gly (40 wt % in relation to the PT + CHT mass) was added to a Petri dish (150 × 85 mm) for solvent evaporation. The unwashed PT/CHT/Gly (UF40) film (approximately 150 × 0.06 mm) was used as a packaging for tomatoes. Commercial polyvinyl chloride was used as a control because it is extensively used as food wrapping. Before packaging, the tomatoes were washed, dried, and cut. The experiment was conducted at room temperature for 18 days. Digital images of the samples were obtained after 0, 9, and 18 days.

### 4.9. Statistical Analysis

The results were statistically analyzed by ANOVA and Tukey’s test, using Prism 8.5 software (GraphPad Software Inc., La Jolla, CA, USA) with a significance level of 5%.

## Figures and Tables

**Table 1:** Water contact angles (WCAs) determined on the films’ surfaces at different contact times.

Films	WCA (°) (*t* = 0 min)	WCA (°) (*t* = 10 min)
UF15	107 ± 6	72 ± 4
UF30	93 ±10	63 ± 2
UF40	112 ± 8	50 ± 3
WF15	109 ± 7	89 ± 8
WF30	93 ± 4	78 ± 5
WF40	95 ± 2	77 ± 4

**Table 2:** Thickness and transparency of the films.

Film	Thickness (mm)	Transparency at λ = 600 nm (%·mm^−1^)
UF15	0.038 ± 0.009	50.35
UF40	0.055 ± 0.014	34.80
WF15	0.028 ± 0.025	68.06
WF40	0.029 ± 0.008	63.64

**Table 3:** Water vapor and oxygen permeability.

Films	Water Permeability (g/m^2^·Day)	Oxygen Permeability (cm^3^/m^2^·Day)
UF40	0.004	1.8
WF15	0.072	0.7

**Table 4:** Experimental conditions used to create the films from polymer blends (30 mL) at a pectin/chitosan (PT/CHT) weight ratio equal to approximately 66/34.

Films	PT Solution (mL)	Gly (wt %)
UF0/WF0 *	20	0
UF5/WF5	20	5
UF10/WF10	20	10
UF15/WF15	20	15
UF20/WF20	20	20
UF30/WF30	20	30
UF40/WF40	20	40

* Condition previously reported in a recent work [24].

## Supplementary Materials

The Supplementary Materials are available online at https://www.mdpi.com/1422-0067/21/22/8663/s1.
