# Supplementary material for: Bactericidal Pectin/Chitosan/Glycerol Films for Food Pack Coatings: A Critical Viewpoint
Source: Int J Mol Sci. 2020 Nov 17;21(22):8663. doi: 10.3390/ijms21228663 (PMC7698469; doi:10.3390/ijms21228663)

Supplementary Information

Fig. S1. Stress-strain curves of the PT/CHT films created from polymer blends at 5, 15, 30 and 40 wt%.

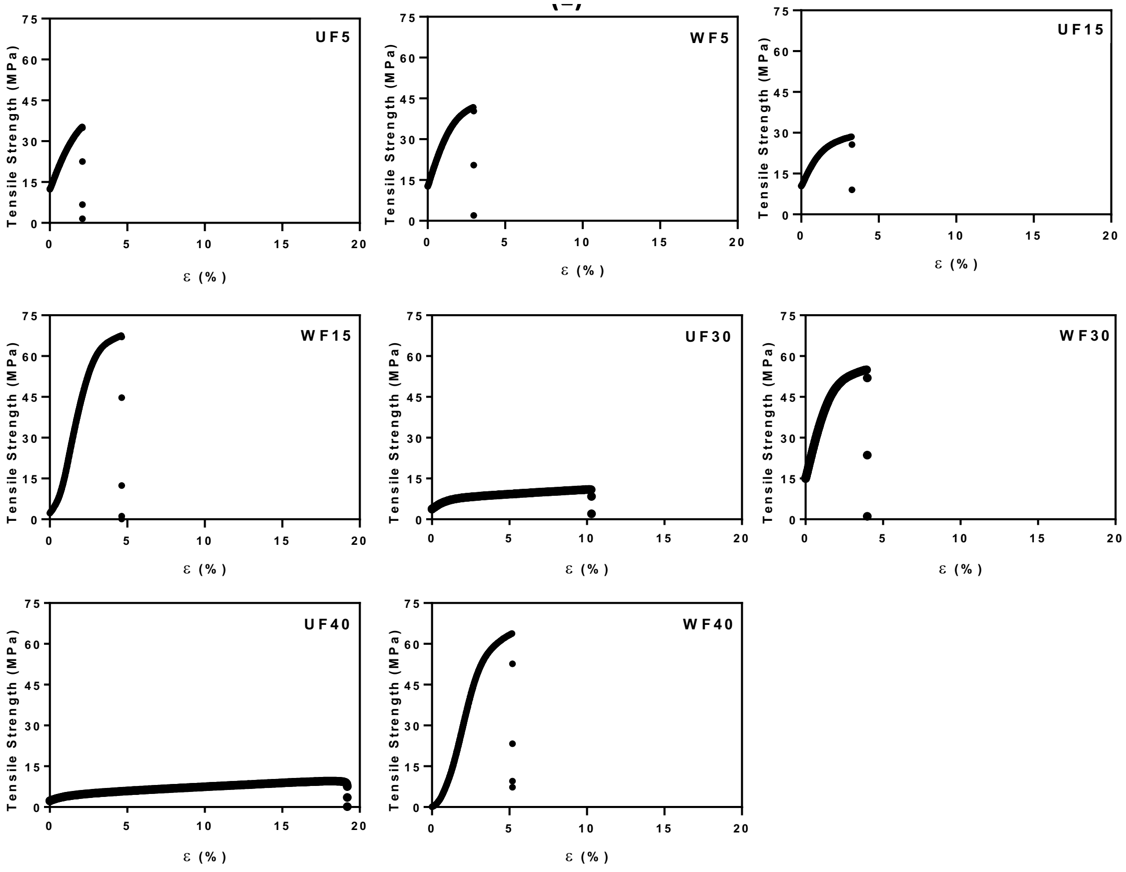

Fig. S2. SEM images of the films.

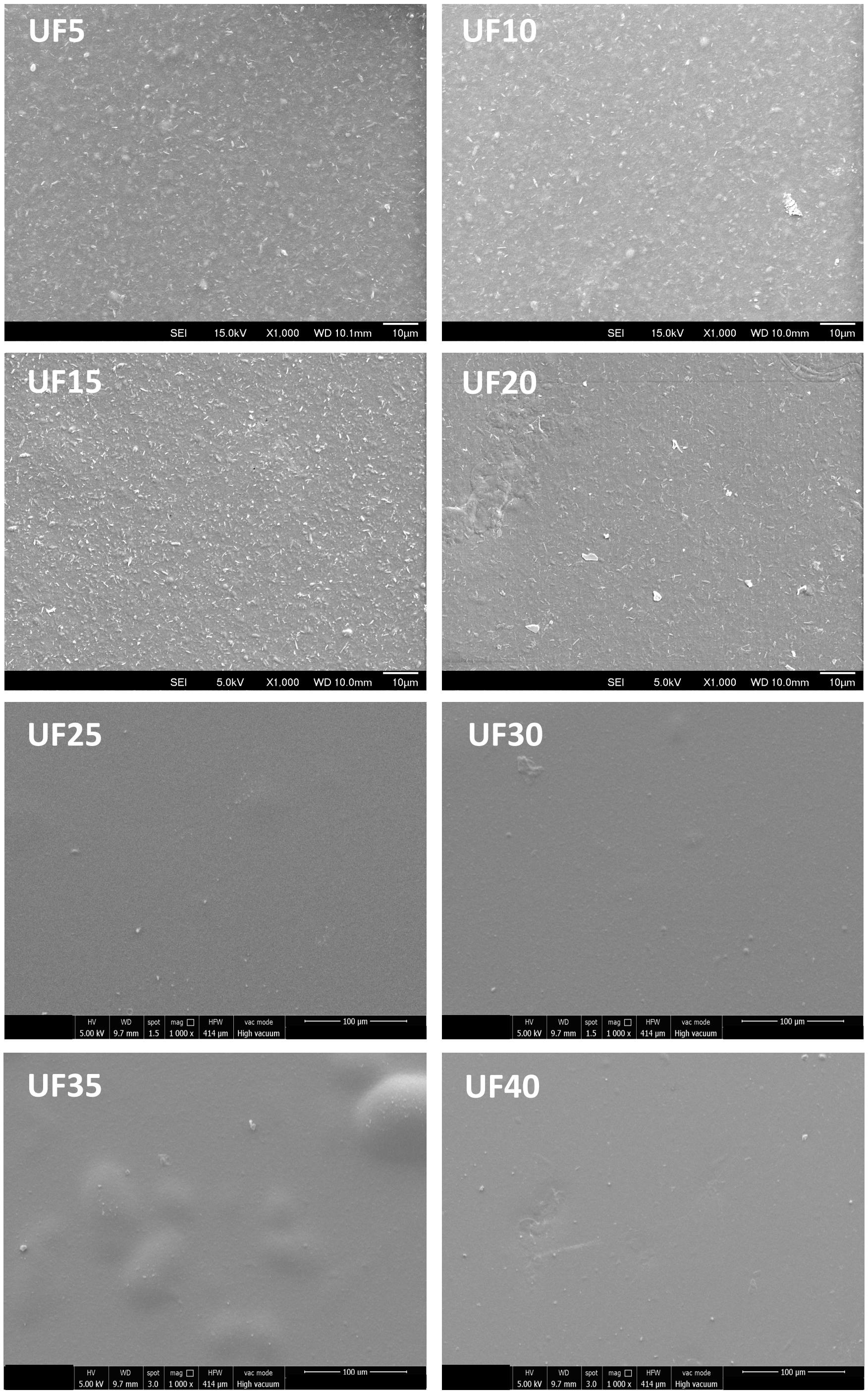

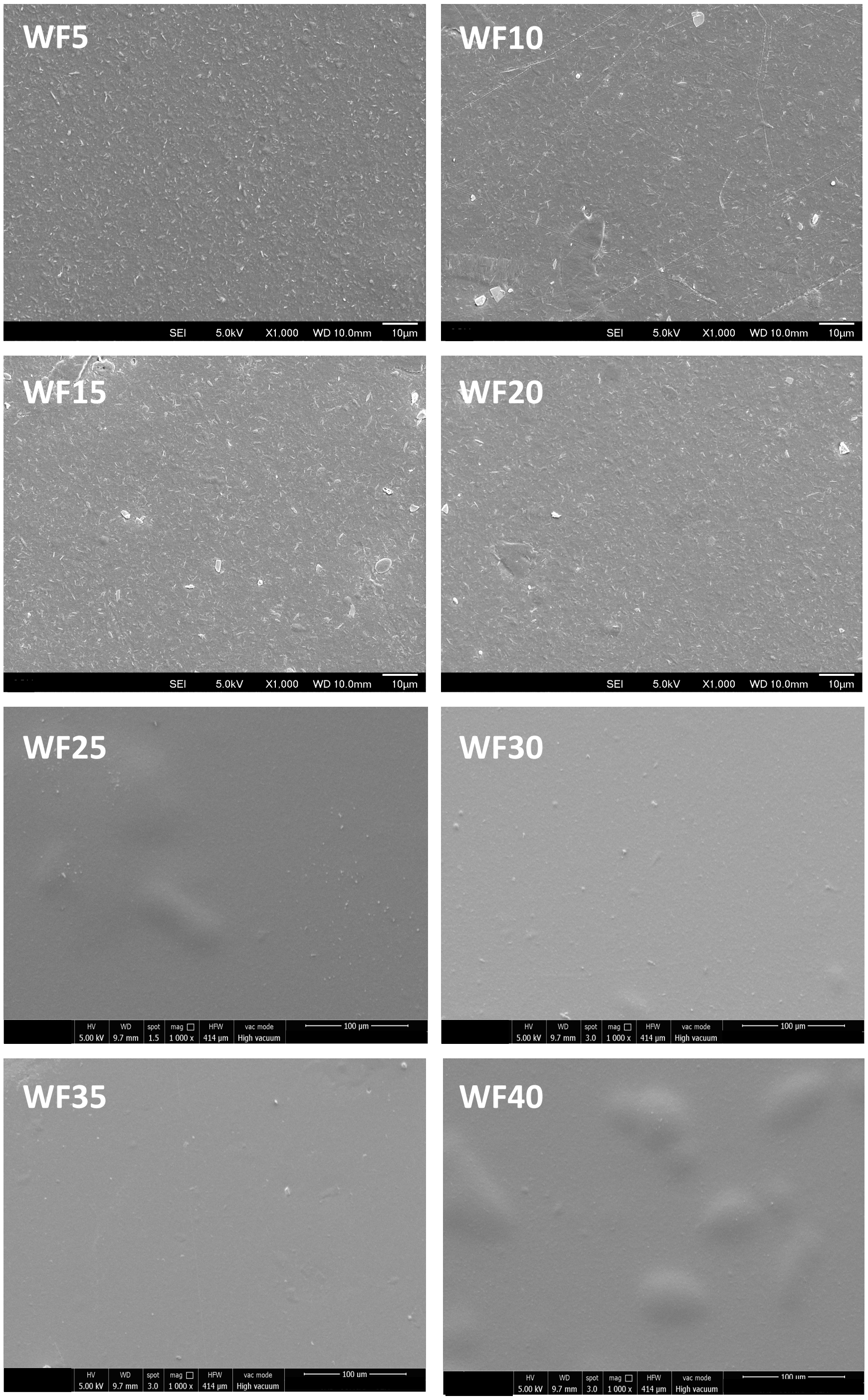
WF5
SEI 5.0kV X1,000 WD 10.0mm 10µm
WF10
SEI 5.0kV X1,000 WD 10.0mm 10µm
WF15
SEI 5.0kV X1,000 WD 10.0mm 10µm
WF20
SEI 5.0kV X1,000 WD 10.0mm 10µm
WF25
HV 5.00 kV WD 9.7 mm spot 1.5 mag 1 000 x HFW 414 µm vac mode High vacuum 100 µm
WF30
HV 5.00 kV WD 9.7 mm spot 3.0 mag 1 000 x HFW 414 µm vac mode High vacuum 100 µm
WF35
HV 5.00 kV WD 9.7 mm spot 3.0 mag 1 000 x HFW 414 µm vac mode High vacuum 100 µm
WF40
HV 5.00 kV WD 9.7 mm spot 3.0 mag 1 000 x HFW 414 µm vac mode High vacuum

Fig. S3. Digital images of one water droplet on the film surfaces after $t = 0$ and $t = 10$ min.

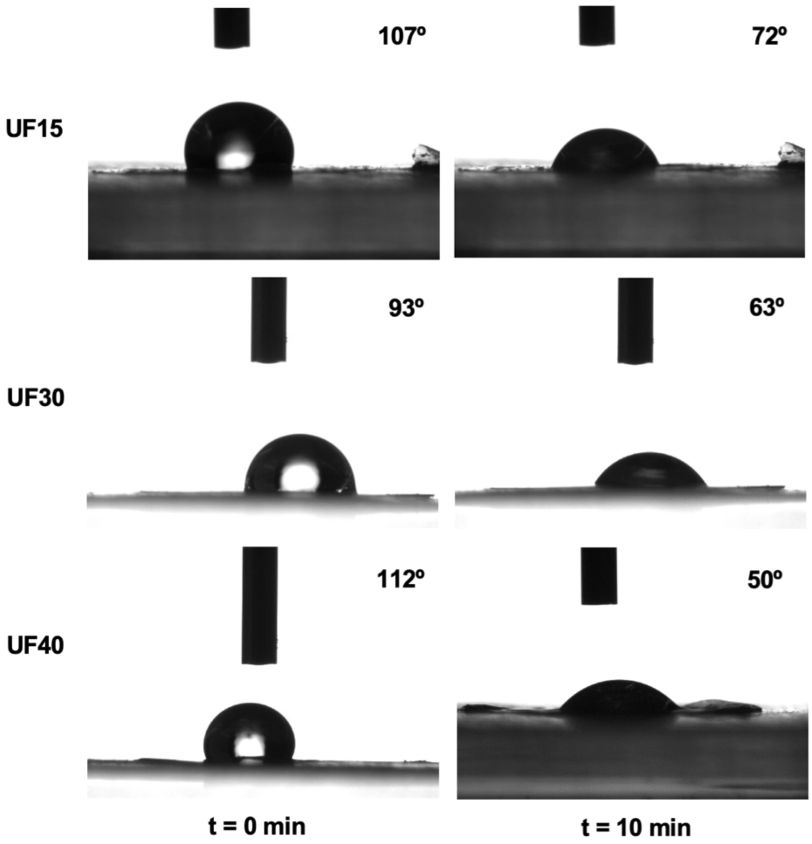

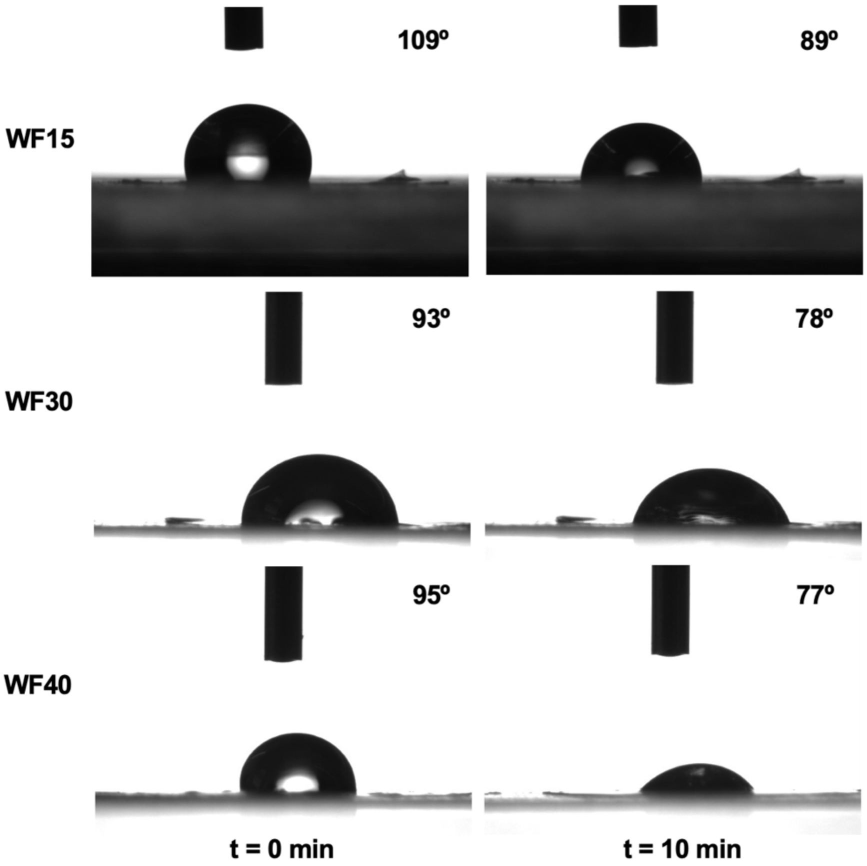

109º
89º
WF15
93º
78º
WF30
95º
77º
WF40
t = 0 min
t = 10 min

Fig. S4. Digital images of film disks seeded on Petri dishes containing *E. coli* cells after 24 h of exposure.

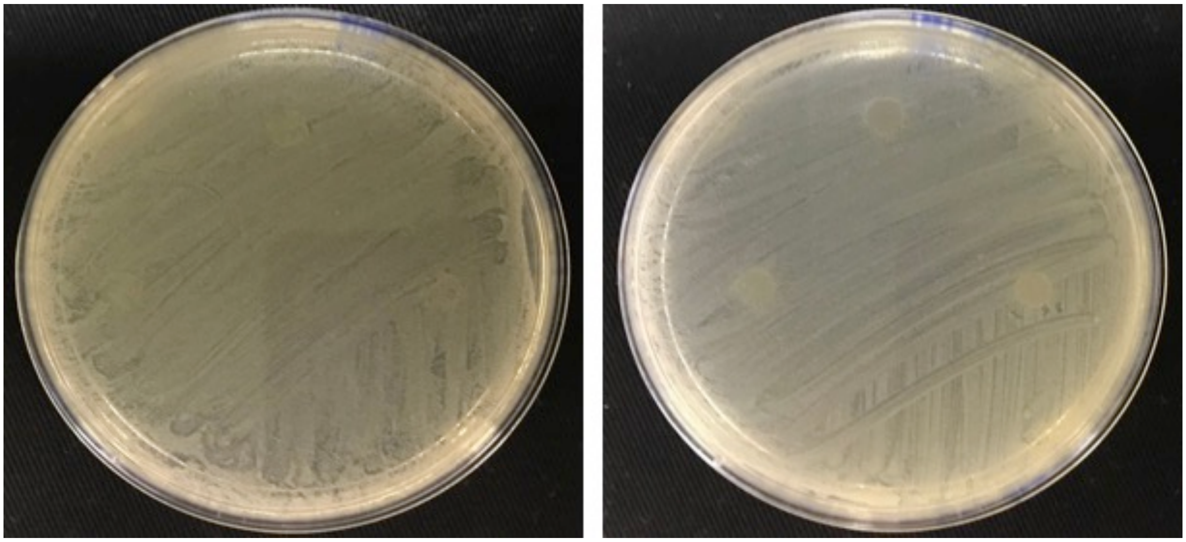

Supplement: Supplementary file 1 [file ijms-21-08663-s001.pdf]
